# Investigations on a clinically and functionally unusual and novel germline *p53* mutation

**DOI:** 10.1038/sj.bjc.6600269

**Published:** 2002-05-03

**Authors:** J Rutherford, C E Chu, P M Duddy, R S Charlton, P Chumas, G R Taylor, X Lu, D M Barnes, R S Camplejohn

**Affiliations:** Richard Dimbleby Department Cancer Research, Guy's, King's and St Thomas' School of Medicine, St Thomas' Hospital, London SE1 7EH, UK; St James's University Hospital, Beckett Street, Leeds LS9 7TF, UK; The General Infirmary, Leeds LS1 3EX, UK; Ludwig Institute, St Mary's Hospital, Norfolk Place, London W2 1PG, UK; ICRF Clinical Oncology Unit, Guy's Hospital, London SE1 9RT, UK

**Keywords:** p53, choroid plexus, Li Fraumeni

## Abstract

This report describes an individual with a rare choroid plexus papilloma in adulthood (age 29) after earlier having an osteosarcoma (age 22). The results from this study, and others, suggest that it may be advisable to consider the possibility of a germline *p53* mutation in adults presenting with choroid plexus tumours. In the current study automated DNA sequencing of genomic DNA detected a novel germline 7 base pair insertion in exon 5 of the *p53* gene in this patient. The alteration in frame would produce amino acid substitutions beginning with alanine to glycine at position 161 and a stop codon at position 182 in the mutated protein. Surprisingly two assays of p53 function gave apparently wild-type results on peripheral blood lymphocytes from this individual. These results led us to carry out more detailed functional tests on the mutant protein. The mutant allele was expressed either at very low levels or not at all in phytohaemagglutinin stimulated lymphocytes. Further, the mutant protein was completely non-functional in terms of its ability to transactivate a series of p53-responsive genes (*p21^WAF1^*, *bax*, *PIG3*), to transrepress a target gene and to inhibit colony growth in transfected Saos-2 cells. However, surprisingly, data from irradiated peripheral blood lymphocytes and transfected Saos-2 cells, suggested that this truncated, mutant protein retains significant ability to induce apoptosis.

*British Journal of Cancer* (2002) **86**, 1592–1596. DOI: 10.1038/sj/bjc/6600269 www.bjcancer.com

© 2002 Cancer Research UK

## 

The tumour suppressor gene *p53* was discovered in 1979 because of its ability to bind to large T antigen (Lane and Crawford, 1979). p53 is a nuclear phosphoprotein that plays a central role in the cellular response to DNA damage by inducing either G_1_ arrest or apoptosis. It functions mainly through its ability to transactivate or repress target genes. Inactivation of wild-type p53 by mutation or interaction with cellular or viral proteins has been found to occur in over 50% of human cancers (Levine, 1997). Li-Fraumeni Syndrome (LFS), which is a rare dominantly inherited cancer predisposition syndrome, is associated with germline *p53* mutations (Malkin *et al*, 1990). Individuals with LFS are at increased risk of developing a large spectrum of cancers, often with a very early onset and multiple primary tumours are common. Primary choroid plexus tumours are rare and usually occur in early childhood. However, several families with LFS have been described, in which there are individuals with choroid plexus tumours, sometimes in young adults (Garber *et al*, 1990). There have been four reports of germline *p53* mutations in families with LFS and childhood choroid plexus tumours (Frebourg *et al*, 1995; Jolly *et al*, 1994; Sedlacek *et al*, 1998; Vital *et al*, 1998). In this report we describe an adult patient with a novel germline 7 base pair insertion in the *p53* gene who presented with an osteosarcoma of the femur at the age of 22 years. This tumour was treated successfully by surgery and chemotherapy. At 29 years, she presented with a choroid plexus tumour which was shown on histology to be an atypical choroid plexus papilloma. There was no unusual family history of cancer (Figure 1

**Figure 1:**
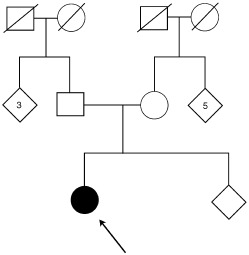
Patients family tree showing no excessive history of cancer. The proband is denoted by the arrow and the numbers (3 and 5) denote the number of unaffected siblings in the respective branches of the family tree.

) and both parents were alive and well in their 50s. Unexpectedly in routine screening this mutation appeared functional in two assays of p53 function (FASAY and apoptotic assay). The FASAY (Flaman *et al*, 1995) is a yeast based assay that looks at the transactivational ability of p53. This assay has been shown to reliably identify both germline (Lomax *et al*, 1997) and sporadic mutations (Duddy *et al*, 2000). The apoptotic assay (Camplejohn *et al*, 1995, 2000), can detect germline p53 mutations by measuring a reduction in the radiation-induced apoptotic response of peripheral blood lymphocytes (PBL) compared with that seen in cells from normal individuals. Due to the unexpected results in these two assays, further functional studies were carried out on this mutation, including an investigation of its ability to induce apoptosis in mammalian cells, to transrepress a target gene and to suppress colony growth.

## MATERIALS AND METHODS

### Sequencing of DNA

Genomic DNA was analysed from peripheral blood leukocytes by direct sequencing of double-stranded PCR products using the Big-Dye terminator kit (PE Applied Biosystems) for exons 5–9. Samples were analysed using a 377 sequencer with 36 cm plates. Sequences were read in both directions. Sequencing of plasmid and cDNA samples was carried out on a ABI 310 sequencer (PE Applied Biosystems).

### Construction of the vectors

For the transfection experiments, the different *p53* mutants (7 base pair insertion, 337C and 344P) were synthesized from wild-type *p53* using the Quikchange site directed mutagenesis kit (Stratagene) and were cloned into the mammalian expression plasmid pC53-SN3. The synthesised mutations were verified using automated sequencing. The 344P (Arg→Pro) mutation is functionally dead, whilst 337C (Arg→Cys) retains partial function and along with wild-type *p53* these two mutants were used as controls in the various assays. The FASAY vectors were kindly provided by Dr R Iggo, Dr JM Flaman and Dr T Frebourg.

### Cell culture and transfection

Saos-2 cells were cultured in DMEM (ICRF Cell Services), supplemented with 4 mM L-glutamine (ICRF Cell Services) and 20% FCS (PAA Laboratories GmbH). Transfections were carried out in 10 cm dishes seeded with 8×10^5^ cells, using 10 μg DNA and the Profection Mammalian Transfection System-calcium phosphate kit (Promega), according to the manufacturer's instructions.

### Functional analysis for the separation of alleles in yeast (FASAY)

The FASAY was carried out on wild-type *p53*, 337C, 344P and the 7 base pair insertion containing plasmids as described by Lomax *et al* (1997). Briefly, the yeast strain yIG397 contains the vector pLS210 which has the selection gene *Ade2* driven by a minimal promoter, CYC1. In the initial FASAY experiments the vector used has three copies of the p53 consensus binding sequence from the ribosomal gene cluster (RGC) immediately upstream of this minimal promoter. Subsequently, yeast containing similar vectors with the p21, bax and PIG3 *p53* promoter binding sites respectively were used (Flaman *et al*, 1998). If the yeast cells are transformed with a wild-type *p53* gene (thus producing wild-type protein) the p53 protein binds to the RGC (or where relevant the *p21^WAF1^*, *bax* or *PIG3*) consensus sequence, transactivating the *Ade2* gene. If, however the *p53* gene has a mutation the resulting p53 protein cannot bind to the relevant consensus sequence and there is no transactivation of the *Ade2* gene. In this case the yeast cells accumulate a red coloured intermediate of the adenine biosynthesis pathway.

### Apoptotic assay

This assay was carried out as described by Camplejohn *et al* (1995). Briefly, PBL were separated from whole blood and cultured for 3 days, at which point half of the PBL were exposed to 4 Gy of radiation. Control and irradiated cells were cultured for a further 24 h, when they were fixed in 70% ethanol. Prior to analysis the samples were acid denatured in 0.1 M HCl (BDH) and stained with propidium iodide (Sigma). The amount of apoptosis was measured by the size of the sub-G_1_ peak by FACS (FACSCalibur, Becton Dickinson).

### Western blotting

For Western blotting, Saos-2 cells were transfected as described and were lysed using NP40 lysis buffer (BDH) after 24 h. The protein concentration was measured using the Bio-Rad DC assay (Bio-Rad Laboratories) and 15 μg of protein denatured in the presence of SDS was loaded onto a 12% SDS–PAGE gel. Proteins were transferred onto a nitrocellulose membrane (Amersham Life Science) and were detected using DO-1 antibody (Santa Cruz Biotechnology). The secondary antibody used was a rabbit anti mouse HRP (DAKO) and this was in turn detected using the ECL Western blotting detection reagent (Amersham Pharmacia Biotechnology).

### Induction of apoptosis in Saos-2 cells

Cells were transfected as described using the calcium phosphate profection kit (Promega). Cells were washed after 16 h and cultured for a further 72 h. The Saos-2 cells were then fixed in 70% ethanol and were stained with propidium iodide (Sigma) and with anti-p53 monoclonal antibody DO-1 (Santa Cruz Biotechnology) for FACS analysis. Apoptosis was determined for both the p53-positive and p53-negative sub-populations by the sub-G_1_ peak size and all samples were compared to the wild-type control.

### Transrepression

5×10^5^ Saos-2 cells were transfected in 60 mm dishes with 1 μg pC53-SN3 (containing wild-type or mutant *p53*) and 5 μg SV40 β-gal. Cells were washed after 16 h and then cultured for 24 h and washed in PBS. 1X reporter lysis buffer (Promega) was used to lyse the cells. We added 20 μl lysate to 150 μl chlorophenolred β-D-galactopyranoside (Boehringer Mannheim) in a 96-well plate, and this was incubated at 37°C. Measurements of absorbance were recorded using a plate reader at 570 nm every 15 min.

### Suppression of colony growth

1×10^5^ Saos-2 cells were transfected in 60 mm dishes with 7 μg pC53-SN3 (containing wild-type or mutant *p53*). Cells were washed after 16 h and were cultured for 48 h. G418 (Calbiochem) at 400 μg ml^−1^ was then added to the media on each dish, and cells were cultured for 4 weeks. Cells were washed in PBS, fixed by adding 70% ethanol, and stained using 0.1% methylene blue. The number of colonies on each plate was counted.

## RESULTS

### Genomic sequencing of DNA

**Figure 2:**
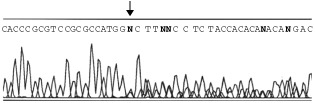
Genomic sequence. Analysis of genomic DNA was carried out from peripheral blood lymphocytes by direct sequencing of double stranded PCR products. A frameshift could be clearly seen starting after nucleotide 13160 in exon 5 (denoted by ↓). The insertion was found to be 7 base pairs in length and was a repeat of the 7 base pair sequence immediately before it. This frameshift would produce amino acid substitutions beginning with alanine to glycine at position 161 and a stop at position 182. Wild-type: CGCGCCATGGCCATCTA; Mutant: CGCGCCATGGGCCATGGCCATCTA

Genomic sequencing of DNA from PBL was carried out in both directions in two independent laboratories from exon 5 through to exon 9. A frameshift mutation, the result of a 7 base pair insertion, was discovered in exon 5 of the *p53* gene, after the first base of codon 161 (Figure 2

). The alteration in frame would produce amino acid substitutions beginning with alanine to glycine at position 161 and a stop codon at position 182 in the mutated protein. The insertion, GCCATGG, was found to be a direct repeat of the 7 base pair sequence immediately upstream in the *p53* gene.

One of the proband's parents elected to have predictive testing and was found not to have the mutation. The other parent declined testing.

### FASAY results

**Figure 3:**
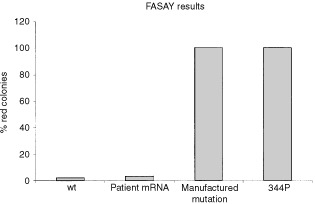
FASAY results. The FASAY gave a normal wild-type result with only 3% red colonies when mRNA was extracted from the patients PHA-stimulated leukocytes. In contrast the 7 base pair mutation synthesised by site directed mutagenesis gave 100% red colonies. The 344P mutation, which is an inactive mutant, also gave 100% red colonies and wild-type p53 gave only 2% red colonies.

The FASAY is a yeast based functional assay, designed to investigate the ability of p53 to transactivate a target gene by binding to a consensus sequence from the ribosomal gene cluster. Wild-type p53 will result in white colonies in the FASAY and mutant p53 will give red colonies. mRNA from the patient's PBL was subjected to RT–PCR and the resulting cDNA was tested in the FASAY. The results show that only 3% red colonies were produced in this experiment, indicating a wild-type result (any value under 10% is considered wild-type). Wild-type p53 gave 2% red colonies, a non-functional mutant (344P) gave a result of 100% red colonies, and a partially functional mutant (337C) resulted in 98% pink colonies; all these results were as expected (Figure 3

). The 7 base pair insertion mutation was then synthesised by site-directed mutagenesis and was put back into the FASAY. Red colonies (100%) were obtained, which indicated that this mutant p53 could not transactivate the *Ade2* gene by binding to the consensus site from the RGC. The difference between the two FASAY results (from PBL and site-directed mutagenesis) could be explained if the patient was expressing the mutant allele either not at all or at only very low levels. In this case the white colonies in the original FASAY using mRNA from the patient's PBL would result exclusively from the wild-type *p53* allele. The engineered mutated cDNA was then put into three more FASAYs, using yeast engineered with different p53 binding sites from the *bax*, *p21^WAF1^* and *PIG3* promoters (Flaman *et al*, 1998). The 100% red colonies were obtained with all three binding sites (data not shown). The mutated p53 could not therefore transactivate *bax*, *p21^WAF1^* or *PIG3* in the FASAY.

### Apoptotic assay

The apoptotic assay (Camplejohn *et al*, 1995) was carried out on PBL obtained from the patient. The assay investigates the ability of these cells to apoptose in response to radiation and measures the increase in apoptosis after exposure to 4 Gy radiation. Unexpectedly, the results showed a normal response with an increase in apoptosis of 46%.

### Western blotting

**Figure 4:**
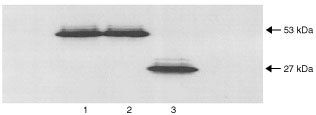
Western blot. The 7 base pair mutation was cloned into the mammalian expression plasmid pC53-SN3, which was transfected into Saos-2 cells. A Western blot was carried out using lysates from these cells, cells transfected with WT p53 and PHA stimulated lymphocytes from the patient. The blot showed WT p53 with the expected size of 53 kDa (lane 1) and the p53 from the patient's lymphocytes also at size 53 kDa (lane 2). The cells transfected with the manufactured mutation showed a protein on the blot at about size 27 kDa (lane 3).

The manufactured 7 base pair insertion mutation was cloned into a mammalian expression plasmid and was transfected into Saos-2 cells. Cell lysates were made of these cells and of Saos-2 cells transfected with wild-type *p53*. PHA stimulated lymphocytes from the patient were also lysed and a Western blot was run using all three lysates (Figure 4

). The blot showed wild-type p53 with the expected size of 53 kDa (lane 1). The lysate from the PHA stimulated lymphocytes also showed a band at 53 kDa (lane 2). However, the Saos-2 cells transfected with the 7 base pair mutation showed the presence of a truncated protein with a size of about 27 kDa. This corresponds to the predicted size of the mutated protein with a stop codon at position 182. The patient's lysate did not contain a band of this size, which again indicated that either the patient was not expressing detectable levels of the mutant allele or that PHA stimulating the lymphocytes favoured the wild-type allele.

mRNA from PHA stimulated lymphocytes was subjected to RT–PCR and the resulting cDNA was sequenced to try to detect the 7 base pair insertion. However, only wild-type sequence was obtained (data not shown).

### Induction of apoptosis

**Figure 5:**
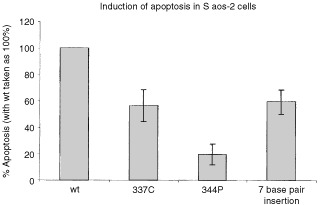
Induction of apoptosis. The pC53-SN3 plasmid containing the p53 insert of interest was transfected into Saos-2 cells, which were washed after 16 h and reincubated for 72 h. The results show that the 7 base pair mutation retained about 65% of the apoptotic ability of WT p53. 344P, the non-functional mutant only retained about 20% apoptotic ability. Statistical analysis showed that the 7 base pair mutation was significantly different from both 344P and WT, with *P*<0.02.

Apoptosis in Saos-2 cells can be induced by the expression of wild-type p53. Saos-2 cells were transfected with the mammalian expression plasmid pC53-SN3, which contained the 7 base pair insertion mutation of *p53*. Controls used included wild-type *p53*, 337C *p53* (a semi-functional mutant) and 344P *p53*. The results showed that the 7 base pair insertion mutation retains 65% of the ability to induce apoptosis compared with wild-type (Figure 5

). The 344P mutation only retained about 20% of the activity of wild-type p53 and the 337C mutant retained about 55%. Statistical analysis using both parametric (paired *t*-test) and non-parametric tests (Fisher's exact test) on these data showed that the 7 base pair insertion mutant was significantly different for apoptosis from both wild-type p53 and the 344P mutant, (*P*<0.02). The 7 base pair mutation was not significantly different from the 337C mutant.

### Transrepression

**Figure 6:**
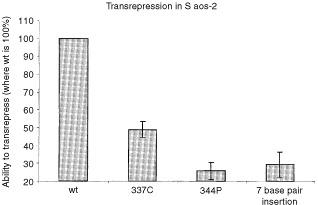
Transrepression. Saos-2 cells were transfected, with pC53-SN3 and pSV40-βgal. Cells were washed after 16 h and lysed after a further 24 h. The results show that the 7 base pair insertion is similar to 344P (*P*=0.33), in that it cannot repress the SV40 promoter, whereas wild-type p53 can.

This method was carried out on the mutants described previously and involved co-transfection of the mammalian expression plasmid pC53-SN3 with a reporter construct consisting of an SV40 promoter upstream of the β-*galactosidase* gene. Wild-type p53 will repress the SV40 promoter and therefore will prevent the expression of β-galactosidase. Addition of a β-galactosidase substrate should produce no colour change. The results obtained in this assay, showed that the 7 base pair insertion mutant and the 344P mutant could not effectively repress the SV40 promoter (Figure 6

). The 337C mutant retained about 50% of the ability of wild-type to repress the SV40 promoter.

### Suppression of growth

**Figure 7:**
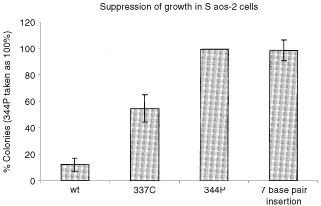
Suppression of growth. Saos-2 cells were transfected with pC53-SN3 as before, but were seeded at only 1×10^5^ cells per 60 mm dish. Cells were washed 16 h after transfection and were treated with G418 at 400 μg ml^−1^ after 48 h. The cells were left for 4 weeks and the number of colonies produced was then counted. The results show that the two mutant proteins (344P and the 7 base pair insertion) produced the most colonies in this experiment. Indeed, the number of colonies was identical for these two mutants. As the 344P mutant is known to be functionally dead, this demonstrates a complete lack of suppression of growth by the 7 base pair insertion mutation.

Wild-type p53 can inhibit the growth of Saos-2 colonies and so a colony forming assay was carried out to determine the ability of the 7 base pair insertion to inhibit growth. Saos-2 cells were again transfected with the pC53-SN3 expression plasmid, in order to observe which of the mutants could suppress the growth of colonies. The results showed that the 7 base pair insertion and the 344P mutants could not suppress the growth of colonies, compared to wild-type p53 (Figure 7

).

## DISCUSSION

The 7 base pair insertion is a novel mutation and is not recorded on the *p53* database. The mutation is suspected to be *de novo*, due to the lack of family cancer history and the lack of mutation in the parent tested. The insertion being so large is unusual, as most reported insertions are between one and three base pairs. Cooper and Krawczak (1993) looked at 20 short insertions, nine of which were single base insertions. Two mechanisms for these insertions were proposed; they could be caused either by slipped mispair mediated by direct repeats (trinucleotide expansions) or mediated by inverted repeats (palindrome). The repeats stabilise a hairpin loop structure. The present reported insertion is a duplication of the previous 7 base pairs and occurs at a palindrome sequence of 8 base pairs.

An association between choroid plexus tumours and LFS has been suggested by others (Garber *et al*, 1990; Yuasa *et al*, 1993). Choroid plexus tumours are rare and are more common in childhood (Dohrmann and Collias, 1975). There have been three previous reports of adult onset choroid plexus tumours in families with multiple malignancies. Two of these were choroid plexus carcinomas, presenting at ages 16 and 27 (Li *et al*, 1988) and one, a choroid plexus papilloma, at age 29 (Faber, 1934). There have been only four previous reports of mutations in *p53* found in families with choroid plexus tumours (Jolly *et al*, 1994; Frebourg *et al*, 1995; Sedlacek *et al*, 1998; Vital *et al*, 1998). However, none of the previously reported mutations were insertions and the patients were all children.

The results from the first two functional assays carried out were normal (FASAY and Apoptotic assay). Further investigations were therefore carried out in order to characterise this mutation. The FASAY using the site-directed mutagenesis 7 base pair insertion cDNA gave an abnormal result (100% red colonies). This result most likely indicated that the mutant allele is expressed at an undetectable level in the original FASAY carried out using mRNA from PHA stimulated PBL from the patient. However, the normal apoptotic response of the patient's cells was surprising as the presence of a non-expressed mutant allele would be expected to result in an abnormal apoptotic response. It is possible that stimulation by PHA of the lymphocytes favoured expression of the wild-type allele hence the wild-type result in the original FASAY. The lymphocytes used in the apoptotic assay are not treated with PHA and it is possible that both alleles were expressed at the very low levels seen normally in unstimulated PBL. This result would then be consistent with the ability of the 7 base pair insertion to induce apoptosis in Saos-2 cells. Western blotting of the patient's PHA stimulated lymphocytes gave a single band present at the expected size of 53 kDa. The transfected Saos-2 cells showed a band at about 27 kDa, which was the size expected for a truncated protein with a stop codon at position 182. No truncated protein was seen in PBL from the patient and sequencing of the mRNA showed no mutation. The induction of apoptosis results showed that the 7 base pair insertion mutation retained 65% of the ability of wild-type to induce apoptosis in Saos-2 cells. However, the mechanism by which a 182 amino acid truncated p53 protein can partially induce apoptosis is not known. The studies on transactivation and transrepression in this report show the 7 base pair insertion mutation to be non-functional. The suppression of colony growth assay also showed the 7 base pair insertion mutation to be clearly inactive. However, in 1995 Haupt and colleagues reported a truncated protein, containing only the first 214 amino-terminal residues of murine p53, which was found to retain its ability to induce apoptosis in HeLa cells, but was transactivationally non-functional. These results are consistent with the findings described here for the 7 base pair insertion mutation. Wild-type p53-induced apoptosis may involve the processes of transactivation or transrepression of target genes. However, an intriguing possibility has arisen following reports that p53 can play a pro-apoptotic role by binding directly to the mitochondrial membrane and interacting with protein members of the bcl-2 family (Marchenko *et al*, 2000). It was suggested by these latter authors that such binding might indeed be the mechanism by which the truncated protein described by Haupt *et al* (1995) induces apoptosis and the same could be true for the 7 bp insertion mutation.

In summary, the 7 base pair insertion mutation is a novel and unusual mutation. The clinical details for the patient were unusual, with the occurrence of a choroid plexus tumour at 29 years. The *p53* mutation found in this individual is clearly functionally abnormal in terms of transactivation and transrepression of target genes and in suppression of colony growth. However, the normal results obtained for apoptosis induction in lymphocytes and the partial ability of the mutant to induce apoptosis in Saos-2 cells implies that a p53 protein with only half of the DNA binding domain present and no oligomerisation domain can retain significant ability to induce apoptosis. In terms of clinical significance it may be advisable to consider the possibility of germline *p53* mutations in adults presenting with choroid plexus tumours since it may influence decisions regarding treatment and imaging.
